# Semi-allogeneic vaccines and tumor-induced immune tolerance

**DOI:** 10.1186/1479-5876-7-3

**Published:** 2009-01-08

**Authors:** Jin Yu, Mark S Kindy, Sebastiano Gattoni-Celli

**Affiliations:** 1 Department of Neurosciences, Medical University of South Carolina, Charleston, SC 29425, USA; 2 Department of Radiation Oncology, Medical University of South Carolina, Charleston, SC 29425, USA; 3 Ralph H. Johnson VA Medical Center, Charleston, SC 29401, USA; 4 SemiAlloGen Inc., 3384 Shagbark Circle, Mt. Pleasant, SC 29466, USA

## Abstract

Experimental results from studies with inbred mice and their syngeneic tumors indicated that the inoculation of semi-allogeneic cell hybrids (derived from the fusion between syngeneic tumor cells and an allogeneic cell line) protects the animal host from a subsequent lethal challenge with unmodified syngeneic tumor cells. Semi-allogeneic somatic cell hybrids were generated by the fusion of EL-4 T lymphoma cells (H-2^b^) and BALB/c-derived renal adenocarcinoma RAG cells (H-2^d^). Cell hybrids were injected intra-peritoneally (i.p.) in C57BL/6 mice (H-2^b^) before challenging the mice with a tumorigenic dose of EL-4 cells. Semi-allogeneic tumor cell hybrids could not form a tumor in the animal host because they expressed allogeneic determinants (H-2^d^) and were rejected as a transplant. However, they conferred protection against a tumorigenic challenge of EL-4 cells compared to control mice that were mock-vaccinated with i.p.-injected phosphate-buffered saline (PBS) and in which EL-4 lymphomas grew rapidly to a large size in the peritoneal cavity. Screening of spleen-derived RNA by means of focused microarray technology showed up-regulation of genes involved in the Th-1-type immune response and in the activation of dendritic antigen-presenting cells (APC). The results of our studies confirm the role of APC in mediating the immune protection induced by semi-allogeneic vaccines by activating a Th-1 response; these studies also reveal that semi-allogeneic vaccines are able to interfere with or even block the tumor-mediated induction of immune tolerance, a key mechanism underlying the suppression of anti-tumor immunity in the immune competent host.

## Background

Almost a century has passed since Paul Erlich first proposed that the immune system has the potential to eradicate cancer even though tumor cells arise from normal cells. Fifty years later the immune surveillance theory put forth that lymphocytes have the capacity to survey and destroy newly arising tumor cells that continuously appear in the body [1]. The conviction that the immune system can be mobilized as well as manipulated to eradicate tumor cells has invigorated the field of tumor immunology, one of the most active fields in immunology. The parallel discoveries of histocompatibility antigens in humans and mice are a good example of how studies in animal models and humans may go hand in hand [2]. In fact, animal studies continue as a basis for important advances because they have allowed the evaluation of multiple parameters in tumor immunology that are not possible in clinical studies [3].

Despite a reasonable understanding of anti-tumor effector mechanisms, clinical studies investigating spontaneous anti-tumor immune responses have yet to lead to reproducible or consistent tumor regression. Thus, the question of why tumors continue to grow and metastasize in immunological competent cancer patients remains unanswered. Several observations have demonstrated that tumors evade and actively suppress the immune system. Tumor evasion of the immune system, termed immune escape, may occur through several mechanisms, including (i) tolerance or anergy induction; (ii) the genetic instability of tumors; (iii) modulation of tumor antigens; and (iv) decreased major histocompatibility complex class I (MHC-I) expression [4]. In addition to evasion of the immune system, tumors actively suppress the immune system directly through production of immune suppressive cytokines and indirectly through the induction of immune inhibitory cells [5]. This secretion of soluble factors is thought to contribute to the Th2-skewed immune responses observed in cancer patients and to induce the development of CD4^+^CD25^+ ^T regulatory cells [6]. The ability of these cells to suppress cytotoxic T lymphocyte (CTL) effector function has been demonstrated in cancer patients [7,8]. This may explain how tumors can indirectly suppress anti-tumor immunity; therefore, immunotherapy modalities aimed at concurrently stimulating anti-tumor immune reactivity, while diminishing tumor-induced immune suppression will be the key to clinical success.

One of the approaches used to increase the immunogenicity of a tumor is called heterogenization, which can be achieved by fusing tumor cells with various allogeneic cells [9,10]. The purpose of heterogenization is to force the host immune response to recognize tumor-associated antigens in the context of allogeneic MHC-I or II molecules or in proximity of strong non-self antigens. The allogeneic/non-self antigen would provide a strong costimulatory signal to enhance anti-tumor immune responses [11]. This approach stemmed from studies with inbred mice and their syngeneic tumors; these studies indicated that the inoculation of semi-allogeneic cell hybrids (derived from the fusion between syngeneic tumor cells and an allogeneic cell line) can protect the animal host from a subsequent lethal challenge with unmodified syngeneic tumor cells [12-14]. We recently reported [15] that semi-allogeneic somatic cell hybrids, generated by the fusion of EL-4 T lymphoma cells (H-2^b^) and BALB/c-derived renal adenocarcinoma RAG cells (H-2^d^), conferred protection against a tumorigenic challenge of EL-4 cells compared to control mice that were mock-vaccinated with phosphate-buffered saline (PBS). Screening of spleen-derived RNA by means of focused microarray technology revealed up-regulation of genes involved in the Th-1-type immune response and in the activation of dendritic antigen-presenting cells (APC). We now report experimental evidence suggesting that, in addition to activating APC and a Th-1-type immune response, semi-allogeneic vaccines also inhibit tumor-induced immune tolerance and anergy.

## Methods

### Cells and semi-allogeneic hybrids

RAG cells are a non-reverting, 8-azaguanine-resistant clone of the Renal-2a cell line, originally derived from a kidney adenocarcinoma of a BALB/c mouse (H-2^d ^haplotype). RAG cells are deficient in the X-linked hypoxanthine-guanine phosphoribosyl transferase gene (HGPRT^-^); therefore, they are killed in culture media containing a supplement of hypoxanthine, aminopterin, and thymidine (HAT). These cells grow as a monolayer. EL-4 cells were established from a T-cell lymphoma induced in a C57BL mouse (H-2^b ^haplotype) by the chemical carcinogen 9,10-dimethyl-1,2-benzanthracene. These cells grow in suspension. RAG and EL-4 cell lines were purchased from the American Type Culture Collection (ATCC). Both cell lines were propagated in Dulbecco's modified Eagle's medium (DMEM) supplemented with 10% fetal bovine serum (FBS), glutamax and antibiotics (Gibco/Invitrogen).

RAG cell monolayers were trypsinized, mixed with EL-4 cells, and fused in 50% polyethylene glycol (PEG)-1450 (cell-culture grade from the ATCC and diluted in serum-free DMEM); after fusion, cells were plated in selective medium (DMEM + 10% FBS and HAT supplement). Under these culture conditions only RAG × EL-4 semi-allogeneic somatic cell hybrids will survive, since RAG cells are killed and EL-4 cells are lost because they grow in suspension and do not attach to the plastic substrate like somatic cell hybrids do. Resulting cell hybrids were propagated in selective medium and used in vaccination animal studies.

### Animals

Pathogen-free C57BL/6 male mice were obtained through the Jackson Laboratories (Bar Harbor, ME). All mice were housed and bred in the VA animal facility located on the seventh floor of the Strom Thurmond Biomedical Research Bldg. After vaccination or mock-vaccination and challenge, mice were monitored very closely for growth of i.p. tumors and sacrificed when their abdomen became clearly extended, generally within three to four weeks. Necropsy was performed on each animal to document the presence of EL-4-derived i.p. tumors. All animal studies were carried out according to the PHS Policy on Humane Care and Use of Laboratory Animals, 2002 and approved by the Ralph H. Johnson VA Medical Center IACUC.

### PCR arrays

Total RNAs were isolated from spleens of mock-vaccinated that developed tumors, and from vaccinated mice that did not develop tumors. These two RNA pools were analyzed for T-cell and B-cell activation (SA Biosciences, cat. # PAMM-053), and for T-cell anergy and immune tolerance (SA Biosciences, cat. # PAMM-074). These analyses combine the multi-gene profiling capabilities of a microarray with the performance of real-time PCR; therefore, the results of the PCR studies are both qualitative and quantitative. The relative or ratio of gene expression, also known as the fold-change or fold regulation, was calculated for each gene using the '2^-ΔΔCt ^method' [16]. To more easily determine the genes that were up-regulated or down-regulated by at least 1.5 fold, a scatter plot comparison was used. Scatter plots compare the normalized, relative expression of each gene2^-ΔCt ^and allow a 'fold-change boundary' to be drawn within the plot. The 'fold-change boundary' segregates the genes up or down regulated based upon the predetermined fold-change value. Scatter plot comparisons were performed by the microarray manufacturer and included only genes that showed either up-regulation or down-regulation by 1.5 fold or more. There are no statistical manipulations within a scatter plot. It simply allows you to visualize the data in a comprehensive fashion.

### Statistical analysis

Data from the animal experiments were analyzed by one-way analysis of variance for analyses of statistical significance, with p < 0.05 indicating statistical significance, using GraphPad Prism software program (GraphPad Software Inc., La Jolla, CA).

## Results and Discussion

### In vivo animal studies

We set to establish the minimum tumorigenic dose of EL-4 cells injected intraperitoneally (i.p.) in C57BL/6 mice (10 mice per group) and tested decreasing numbers of EL-4 cells (1 × 10^4^, 5 × 10^3^, 2 × 10^3^, 1 × 10^3^, 5 × 10^2^, and 2 × 10^2 ^per mouse, respectively) in PBS (0.2 mL per mouse). We found that, in these experimental conditions, 1 × 10^3 ^EL-4 cells were very close to the minimum tumorigenic dose for C57BL/6 mice, most of which developed abdominal tumors within three to four weeks. Even at 2 × 10^2 ^cells per mouse we observed tumor formation, a clear evidence of the highly malignant phenotype of these cells.

**Figure 1:**
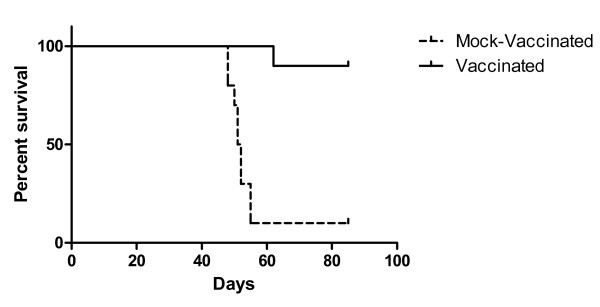
**Survival of vaccinated vs. mock-vaccinated mice**. Ten C57BL/6 male mice were vaccinated i.p. with 1 × 10^6 ^RAG × EL-4 semi-allogeneic somatic cell hybrids [irradiated with 30 Gy (3,000 rad) in a ^137^Cs irradiator]. As a control, ten age-matched mice were mock-vaccinated i.p. with 0.5 mL PBS. Four weeks after vaccination or mock-vaccination each mouse was challenged by i.p. injection with 1 × 10^3 ^EL-4 and mice were monitored daily for ten more weeks. Mice with enlarging abdominal tumors were euthanized and the presence of tumor was confirmed at necropsy (P < 0.0001 between the two survival curves).

Subsequently, we set to investigate whether irradiated RAG × EL-4 semi-allogeneic somatic cell hybrids could protect C57BL/6 mice from a lethal challenge with 1 × 10^3 ^EL-4 cells. Ten-week-old C57BL/6 male mice were injected intraperitoneally (i.p.) with 1 × 10^6 ^RAG × EL-4 semi-allogeneic somatic cell hybrids [irradiated with 30 Gy (3,000 rad) in a ^137^Cs irradiator] in 0.5 mL PBS. As a control, age-matched mice were mock-vaccinated i.p. with 0.5 mL PBS. Four weeks after vaccination or mock-vaccination each mouse was challenged by i.p. injection with 1 × 10^3 ^EL-4 cells in 0.2 mL PBS. Figure 1 shows that less than four weeks after challenge, nine of ten mock-vaccinated mice had to be euthanized because of large abdominal tumors (verified at necropsy); in contrast, only one of ten mice vaccinated with irradiated RAG × EL-4 semi-allogeneic somatic cell hybrids had to be euthanized almost five weeks after challenge, because of a large abdominal tumor. No further changes were observed at ten weeks after challenge, considered a safe time-frame for measuring established anti-tumor protection. We have performed several experiments of vaccination followed by challenge, obtaining comparable results (complete protection from tumor at more than ten weeks after challenge).

### Studies with PCR arrays

Total RNAs were isolated from spleens of mock-vaccinated that developed tumors, and from vaccinated mice that did not develop tumors. We purified RNA from protected mice at ten weeks after challenge, under the assumption that those mice had true immune protection against the EL-4-derived tumor. Obviously, we had to purify RNA from the spleen of tumor-bearing mice much earlier (before they would die). These two RNA pools were analyzed for T-cell and B-cell activation (SA Biosciences, cat. # PAMM-053), and for T-cell anergy and immune tolerance (SA Biosciences, cat. # PAMM-074). The results of these experiments confirmed to a large extent what we reported previously [15], including the enhanced expression of CD80 and CD86. However, the transcriptomic profile of genes associated with T-cell anergy and immune tolerance yielded the most informative results. The value of these microarray studies also stems from the fact that it combines the multi-gene profiling capabilities of a microarray with the performance of real-time PCR; therefore, the results of the microarray studies are both qualitative and quantitative. Table 1 shows the summary of these analyses for genes that were either over-expressed or down-regulated at the transcription level.

**Table 1:** Differential expression of genes involved in T-cell anergy and immune tolerance.

**GENE**	**FUNCTION**	**Fold UP-Regulation** **(DOWN-Regulation)**
**BTLA**	Induced during activation of T cells; Expressed on Th1 cells; Interacts with B7 homolog B7H4.	**3.0; 2.7**

**CD40**	Co-stimulatory molecule expressed by B cells, dendritic cells, and follicular dendritic cells.	**2.6; 2.7**

**CD40L**	Expressed by activated T cells; Binds to CD40 on APC.	**1.6, 2.1**

**CD70**	Expressed by activated T and B cells; Induces proliferation of co-stimulated T cells; Enhances the generation of CTLs.	**3.0, 1.7**

**FASLG**	Interacts with FAS and triggers apoptosis.	**2.1, 2.2**

**GZMB**	Granzyme B is crucial for apoptosis of target Cells by CTLs.	**3.2, 2.4**

**HDAC9**	Histone deacetylase 9, transcriptional repressor.	**3.4, 2.7**

**ICOS**	Inducible T-cell co-stimulator.	**2.2, 1.7**

**IFNG**	Th1- and dendritic cell-specific cytokine.	**5.5**

**LTA**	Lymphotoxin α or tumor necrosis factor β.	**2.1, 1.7**

**PRF1**	Perforin, key CTL effector molecule.	**2.4, 2.6**

**TBX21**	Th1-specific transcription factor that controls the expression of IFN-γ.	**2.6**

**TNFRSF4**	Receptor involved in CD4+ T cell response.	**2.0, 2.1**

**TNFSF10**	TNF-like cytokine; Induces apoptosis of tumor cells.	**2.1, 1.6**

**TNFSF8**	TNF-like cytokine; Induces apoptosis of some lymphoma cells.	**2.1, 1.7**

**CCR4**	Receptor for CC chemokines.	**(2.9, 2.0)**

**GATA3**	Transcription factor that favors expression of Th2-type cytokines.	**(2.4, 1.5)**

**IL5**	Cytokine for growth and differentiation of B cells and eosinophils.	**(1.3, 4.1)**

**IL6**	Inhibits T cell activation; Inhibits the CD40L system; Induces a Th2-type cytokine response.	**(5.9, 6.7)**

**LAT**	Required for TCR-mediated signaling; Possibly associated with overstimulation and apoptosis of T cells.	**(2.8, 2.1)**

**PDCD1**	Induction and maintenance of T-cell tolerance.	**(2.7, 2.0)**

**RNF128**	Involved in induction of anergic phenotype	**(5.4, 4.5)**

**TNFRSF8**	Positive regulator of apoptosis; Limits proliferation of CD+ effector T cells.	**(4.7, 2.8)**

Expression was measured by real-time RT-PCR. Total RNA was purified from splenocytes of vaccinated immune mice and compared to RNA from control, non-immune mice.

These studies were undertaken to further our understanding of the mechanisms underlying the specific anti-tumor response induced by semi-allogeneic vaccines. The results of our animal studies and PCR array experiments confirm that semi-allogeneic vaccines trigger the activation of dendritic APC and CTL to specifically recognize and kill their target tumor cells. These studies also reveal that semi-allogeneic vaccines are able to interfere with or even block the tumor-mediated establishment of immune tolerance, a key mechanism underlying the suppression of anti-tumor immunity in the immunocompetent host. The results reported in this short communication represent an additional building block for future studies aimed at assessing, by fluorescence-activated cell sorting (FACS), the phenotypic profile of splenocytes of vaccinated and mock-vaccinated mice at various time points before and after vaccination and/or challenge. Furthermore, we plan to undertake functional analysis of splenocyte subsets to corroborate, by intracellular staining, the results of the microarray studies and document the differential expression of select proteins and cytokines.

## Competing interests

MSK and SCG have interests in SemiAlloGen.

## Authors' contributions

JY was responsible for conducting the animal experiments, MSK designed the animal experiments and performed the statistical analysis, and SGC was responsible for overall experimental design and wrote the manuscript. All authors read and approved the final manuscript.
